# A pulsatile release platform based on photo-induced imine-crosslinking hydrogel promotes scarless wound healing

**DOI:** 10.1038/s41467-021-21964-0

**Published:** 2021-03-15

**Authors:** Jian Zhang, Yongjun Zheng, Jimmy Lee, Jieyu Hua, Shilong Li, Ananth Panchamukhi, Jiping Yue, Xuewen Gou, Zhaofan Xia, Linyong Zhu, Xiaoyang Wu

**Affiliations:** 1 The University of Chicago, Ben May Department for Cancer Research, Chicago, IL 60637 USA; 2 Key Laboratory for Advanced Materials and Joint International Research Laboratory of Precision Chemistry and Molecular Engineering, Feringa Nobel Prize Scientist Joint Research Center, School of Chemistry and Molecular Engineering, East China University of Science & Technology, 130 # Meilong Road, Shanghai, 200237 China; 3 Burns Center of Changhai Hospital, Shanghai, China

**Keywords:** Biomaterials, Skin diseases

## Abstract

Effective healing of skin wounds is essential for our survival. Although skin has strong regenerative potential, dysfunctional and disfiguring scars can result from aberrant wound repair. Skin scarring involves excessive deposition and misalignment of ECM (extracellular matrix), increased cellularity, and chronic inflammation. Transforming growth factor-β (TGFβ) signaling exerts pleiotropic effects on wound healing by regulating cell proliferation, migration, ECM production, and the immune response. Although blocking TGFβ signaling can reduce tissue fibrosis and scarring, systemic inhibition of TGFβ can lead to significant side effects and inhibit wound re-epithelization. In this study, we develop a wound dressing material based on an integrated photo-crosslinking strategy and a microcapsule platform with pulsatile release of TGF-β inhibitor to achieve spatiotemporal specificity for skin wounds. The material enhances skin wound closure while effectively suppressing scar formation in murine skin wounds and large animal preclinical models. Our study presents a strategy for scarless wound repair.

## Abstract (web summary)

Dysfunctional and disfiguring scars can result from aberrant wound repair. Here, the authors develop a wound dressing material based on an integrated photo-crosslinking strategy and a microcapsule platform with pulsatile release of TGF-β inhibitor to achieve spatiotemporal specificity for scarless wound repair.

## Introduction

Skin wound healing is a dynamic and interactive process involving the collaborative efforts of growth factors, extracellular matrix (ECM), and different tissue and cell lineages^1–3^. Wound repair in skin involves four different stages: hemostasis, inflammation, re-epithelization, and resolution/tissue remodeling^1–3^. Although both ectodermally derived epidermis and the mesodermally derived dermal tissue of skin have strong regenerative potential upon wounding, dysfunctional and disfiguring scars can result from the failure of the wound to properly transition from the regenerative stage to the resolving stage^4–9^. Skin scarring involves excessive deposition and misalignment of ECM, increased wound cellularity, and chronic inflammation pathologies. Scarring in the skin after trauma, surgery, burn, or other injury presents a significant medical problem, often leading to loss of tissue function, restriction of movement, and severe psychological morbidities^4–9^. Although accumulating studies with a range of different model systems have increased our understanding of the cellular and molecular basis underlying skin scar formation, they have not been effectively translated to therapy. As a result, current treatments and preventive measures against scarring remain unreliable. Development of effective therapeutic approaches for skin scar management is urgently needed.

Transforming growth factor-β (TGFβ) signaling controls a wide variety of cellular processes, including proliferation, differentiation, tissue homeostasis, and regeneration^10–13^. TGFβ has been well-recognized as a key regulator of skin wound repair^7,14–16^. It exerts pleiotropic effects on different phases of wound healing by regulating proliferation of epidermal and dermal cells, epithelial cell migration, ECM production, and the immune response. Dysregulated TGFβ signaling contributes heavily to pathological skin scarring. It has been shown that three isoforms (1-3) of TGFβ may have different temporal effects on skin wound repair and scar formation, and disruption of their expression may lead to hypertrophic scarring^14,15,17–23^. Expression and signaling of TGFβ1 and β2 are upregulated in hypertrophic scars both in vivo and in vitro. Skin scar tissue also exhibits persistent expression of *TβRI* (TGFβ receptor I) and *TβRII* (TGFβ receptor II), whereas their expression usually declines during the tissue remodeling process in normal skin wound healing^14,15,17^. Thus, the TGFβ pathway has been considered as a promising therapeutic target for treatment of aberrant skin wound repair and scarring. Earlier works of Ferguson and colleagues have shown that neutralizing antibodies against TGFβ1/2 can significantly reduce skin scarring in rodent models^24^. Recombinant TGFβ3 (avotermin) has also been tested as a prophylactic prevention for scar formation following surgical procedures^25^. However, the role of TGFβ signaling in skin wound repair is complex. Although blocking TGFβ signaling has been shown to reduce fibrosis in a number of animal models^8,10,21^, it may also lead to chronic or non-healing wounds^26–29^. Interestingly, it has been shown that timing of TGFβ signaling inhibition is critical for therapeutic effect. Administration of neutralizing antibodies at an early stage before completion of re-epithelization may impair cutaneous wound healing without improvement of scarring. By contrast, treatment at a resolution stage of wound healing (usually 6-13 days post wounding) can lead to significant improvement of skin scarring^30^. As a central signaling cascade involved in many cellular processes, systemic inhibition of TGFβ activity may lead to significant side effects^10,31,32^. Current advances in drug delivery systems represent a great opportunity to develop therapeutic strategies with spatiotemporal specificity for skin wound repair^33^.

In this study, we devise a pulsatile drug-release platform to achieve scarless wound healing by combining a sutureless wound closure hydrogel material^34–36^ with a biodegradable microcapsule system that can be tailored to release its contents in skin wounds at the desired time. Microcapsules fabricated with different approaches have been tested for drug delivery^37–43^. However, most microcapsules will release their contents in a continuous manner^42,43^. To address this issue, we develop a water-oil-water double emulsion strategy to encapsulate proteins within a photo-crosslinkable poly-lactic-co-glycolic acid (PLGA) shell, which can produce microcapsules with pulsatile drug release kinetics after administration. When integrated into the wound via in situ photo-inducible imine-crosslinking hydrogels^34–36^, the capsules can take part in forming a stable and uniform microdopant packing in the wound area. Our results further show that pulsatile release of the TGF-β inhibitor can accelerate skin wound closure while suppressing scarring in murine skin wounds and large animal preclinical models, suggesting that it could be an effective approach to achieve scarless wound healing in skin.

## Results

### Fabrication of PLGA microcapsules

**Fig. 1:**
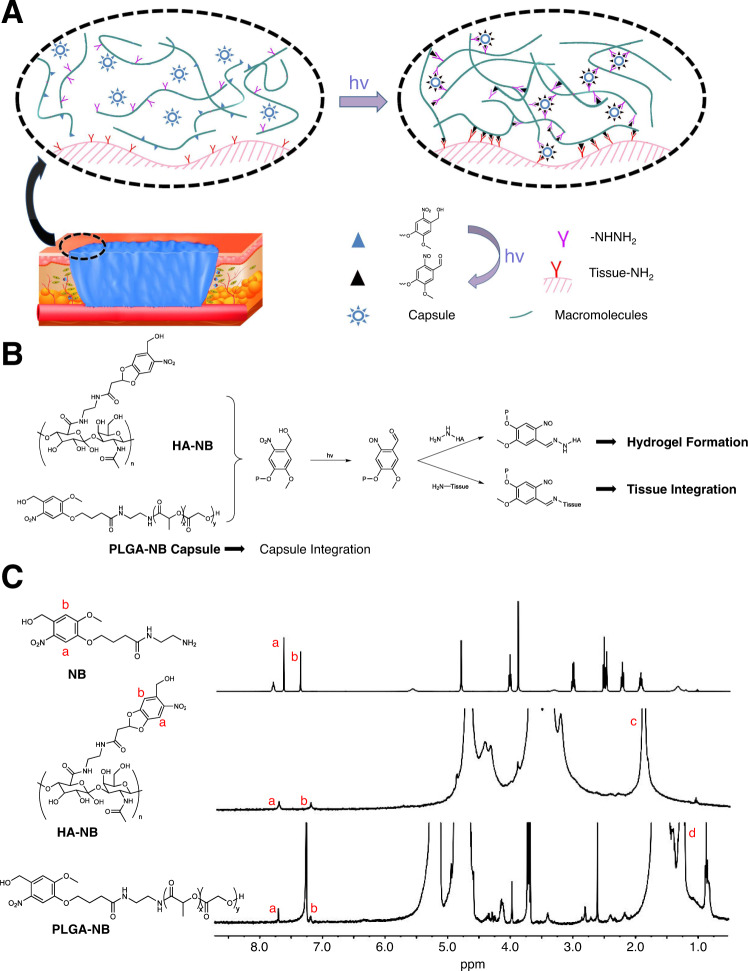
Engineering PLGA microcapsules that can be integrated to skin wounds through photo-inducible crosslinking. **A** Schematic illustration of photo-induced crosslinking mechanism for tissue integration of hydrogel and PLGA-NB capsules. **B** Schematic illustration of the chemical structures of HA-NB and PLGA-NB, as well as photo-inducible integration of microcapsules with hydrogel and wounded tissue. **C** Structure characterization of NB, HA-NB, and PLGA-NB by ^1^H NMR (400 MHz).

PLGA is a promising material for drug delivery, owing to its superior biocompatibility and biodegradability^44^. However, a key challenge for wound repair is the delivery approach for drug carriers, as skin wounds represent a highly dynamic and complex environment, and there are very few approaches, surgical or otherwise, which can effectively retain and protect drug-carrying capsules within the wound. An imine-based photogelation process can achieve intimate biomaterial-tissue integration and enhance skin wound healing in vivo^34–36^. To fabricate PLGA-based capsules that can be integrated into a photo-crosslinking hydrogel, we grafted *o*-nitrobenzene (NB) to PLGA to obtain photo-responsive polymer PLGA-NB (NB-modified poly(lactic-co-glycolic acid), MW = 50 kDa, lactic acid: glycolic acid =75:25) via amide linkage in the assistance of 1-ethyl-3-(3-dimethylaminopropyl) carbodiimide hydrochloride and N-hydroxysuccinimide (Fig. 1A, B and Supplementary Fig. 1). As an excellent tissue-integratable platform, we chose HA-NB (NB-modified hyaluronic acid, MW = 340 kDa, 3.5% substitution degree of NB molecule) and HA-CDH (carbohydrazide-modified hyaluronic acid, MW = 48 kDa, 10.8% substitution degree of hydrazide groups) as pre-gelling polymers to deliver PLGA-NB capsules (Supplementary Fig. 1). The ^1^H NMR spectrum of PLGA-NB and HA-NB exhibits characteristic peaks that represent the benzene hydrogen atoms (a, b) in NB moiety (Fig. 1C).

**Fig. 2:**
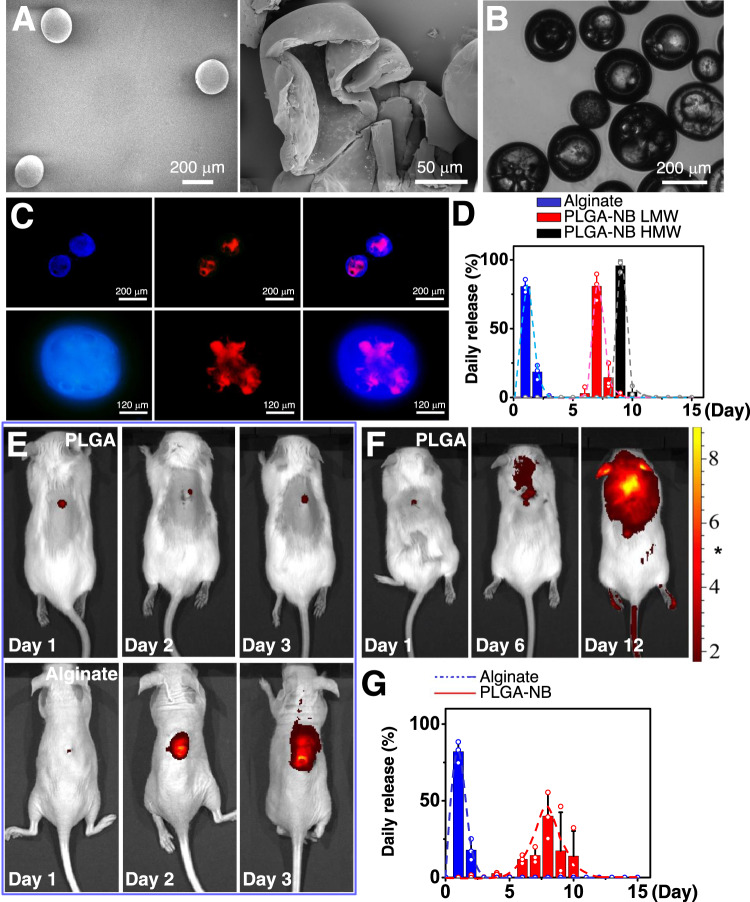
PLGA-NB capsules exhibit delayed and pulsatile cargo release in vitro and in vivo. **A** FESEM images of the intact (left) and crushed (right) PLGA-NB capsules. **B** Phase contrast image (light microscope) of PLGA-NB capsules. **C** Epifluorescence imaging of PLGA-NB capsules loaded with rhodamine-conjugated BSA. **D** Daily release of BSA in vitro with alginate capsules, low molecular weight (LMW), or high molecular weight (HMW) PLGA-NB capsules. *n* = 3 independent samples. Data are presented as mean ± SD. Error bars represent SD (standard deviation). **E** Intravital fluorescence imaging of animals 1–3 days after subcutaneous injection of control (alginate) or PLGA capsules loaded with DiI/DiD. **F** Intravital fluorescence imaging of animals 1–12 days after subcutaneous injection of PLGA capsules loaded with DiI/DiD. **G** Quantification of daily cargo release in vivo for alginate and PLGA capsules. *n* = 3 independent samples. Data are presented as mean ± SD. Error bars represent SD. Source data are provided as a Source Data file.

PLGA microcapsules fabricated with traditional approaches have inherent caveats, including low encapsulation efficiency and an initial high burst release of cargo^44,45^, making it unsuitable as a vehicle for timed-release of TGFβ regulators. To resolve these issues, we employed water–oil–water (W/O/W) double emulsion strategy to prepare PLGA-NB capsules with increased shell thickness to suppress initial burst release and a hollow structure to enhance encapsulation of hydrophilic drug/proteins. High-resolution Field-Emission Scanning Electron Microscopy (FESEM) was used to assess the size and structure of the PLGA-NB capsules (Fig. 2A). The fabricated capsules are spherical capsules with smooth surfaces free of pores and cavities. We next crushed the capsules to examine the internal structure with FESEM. Our data show that the PLGA-NB capsules contain a hollow inner structure with a single-core (Fig. 2A). The aqueous droplets that were entrapped in the interior of the capsules evaporated during the drying process and formed hemispherical pits on the inner wall. The size of the capsule was determined by both light microscopy (Fig. 2B) and FESEM. The diameter of the capsules was 220 ± 20 μm and the thickness of the wall was 20 ± 3 μm. When examined under fluorescence microscopy, the shell of PLGA-NB capsules emitted strong blue fluorescence, whereas capsules derived from PLGA alone did not exhibit any fluorescent signals, which confirms successful modification of PLGA with NB. When loaded with fluorescently labeled BSA, the core of the PLGA-NB capsules shows fluorescent signals with the expected spectrum (Fig. 2C).

To determine the biocompatibility of the capsules, empty capsules and capsules loaded with control protein or test drugs were incubated with Caco-2 or HeLa cells at different concentrations. All capsules showed no significant cytotoxicity in vitro at various concentrations (Supplementary Fig. 2).

### Drug release kinetics of PLGA microcapsules

To assess the drug release kinetics, we first examined the release profile in vitro of the capsules fabricated from PLGA with different molecular weight. Serving as a control, alginate-derived microcapsules released over 95% of the encapsulated cargo (BSA protein) within the first 2 days (Fig. 2D). By contrast, BSA remained undetectable in the solution for 6 days or 8 days when encapsulated in PLGA microcapsules prepared from low molecule weight (LMW, 50,000 Kd) or high molecular weight (HMW, 80,000 Kd) precursors, respectively. LMW PLGA capsules exhibited strong and pulsatile release of BSA during day 6-8, whereas the HMW capsules released BSA during day 9–10 (Fig. 2D). These results confirmed that the core-shell structure of our capsules can lead to delayed and pulsatile release of drugs with negligible initial burst release, and increase of PLGA molecular weight can prolong the time lag for drug release.

Drug release kinetics in vivo can be affected by protein denaturation, hydrolysis, tangles, noncovalent aggregation, and adsorption^44,45^. To evaluate cargo release profile in vivo, we encapsulated a mixture of fluorescence dyes, DiI/DiD (1,1′-dioctadecyl-3,3,3′,3′-tetramethylindodicarbocyanine perchlorate) in LMW PLGA capsules. Due to forster resonance energy transfer (FRET), the fluorescence of DiI (donor fluorophore) can be significantly quenched when loaded inside the capsules, and release from the carriers can alleviate the quenching and greatly enhance its fluorescence intensity^46^. When injected subcutaneously to CD1 mice, control alginate capsules released the fluorescence dye within 2 days, whereas PLGA capsules discharged its cargo after 6 days (Fig. 2E and F). No significant increase in fluorescence was detected in PLGA group during the first 5 days (Fig. 2G). It is noteworthy that release of DiI/DiD seems to be slower in vivo, likely due to the complexity of tissue environment in vivo^47,48^. The release took ~5 days in vivo, and peaked at Day 8 (Fig. 2G). Together, our results strongly suggest that the PLGA carrier can mediate the timed release of cargo in vivo, making it an ideal candidate to deliver TGFβ regulators for skin scarring treatment.

### Preparation and characterization of capsule-loaded hydrogels

**Fig. 3:**
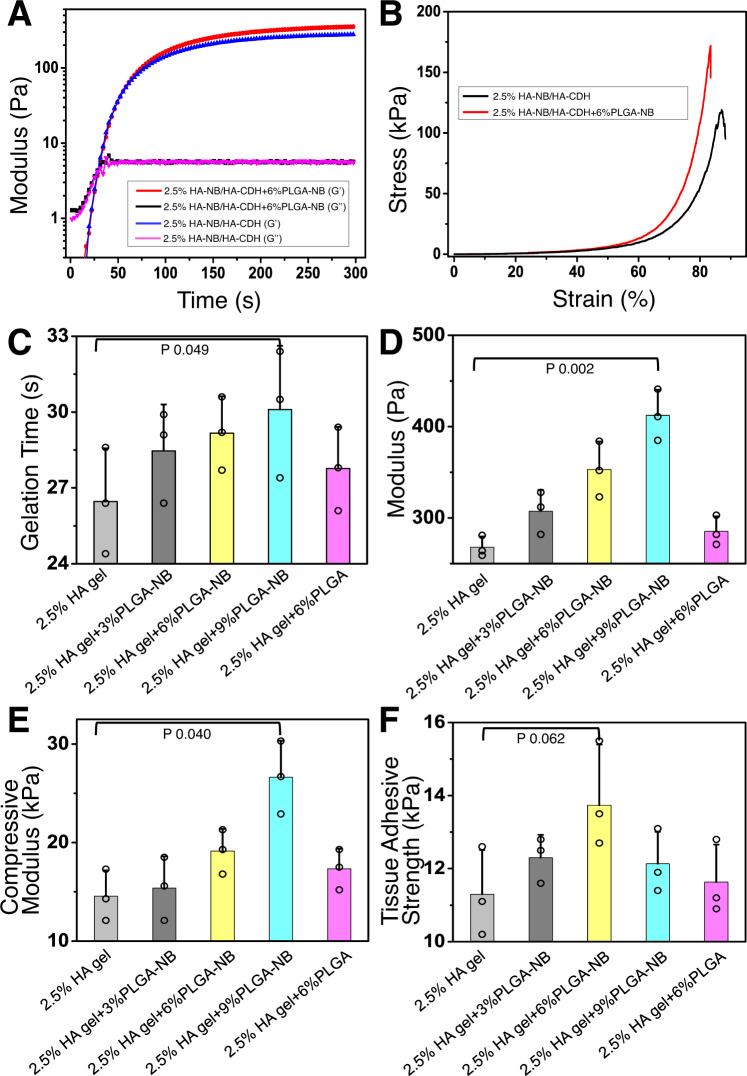
PLGA-NB capsules can strengthen the photo-crosslinking hydrogel network. **A** Rheology analysis demonstrates the hydrogel formation for HA-NB/HA-CDH hydrogel with or without PLGA-NB capsules. **B** Stress measurement for HA-NB/HA-CDH hydrogel with or without PLGA-NB capsules. (**C**–**F**) Gelation time, modulus, compressive modulus, and tissue adhesive strength measurement all demonstrate that PLGA-NB capsules can strengthen and enhance the HA-NB/HA-CDH hydrogel. *n* = 3 independent samples. Data are presented as mean ± SD. All error bars represent SD. p values for representative data are shown (One-tailed paired t-test). Source data are provided as a Source Data file.

Skin wounds represent a highly dynamic and complex environment, requiring a safe and robust delivery approach for drug carriers. HA-NB and HA-CDH hydrogel are photo- crosslinkable, and can generate mechanically strong hydrogel^34–36^ to effectively retain and protect drug-carrying capsules in the skin wounds (Fig. 1A). The material is bio-compatible and degradable. It can be cleared from skin wounds 2 weeks after application^36^. The photogelation processes were investigated by the dynamic rheological tests. As shown in Fig. 3A and B, both the G′ and G″ increased as the irradiation time was increased, indicating the imine crosslinking between photogenerated aldehyde groups and hydrazide groups in the backbone of HA. For HA-NB/HA-CDH gels, the gelation time was approximately 27 s and the final storage modulus was approximately 270 Pa (Supplementary Table 1). When PLGA-NB capsules were introduced to the HA-NB/HA-CDH gels, the final storage modulus was increased to approximately 350 Pa with an approximately 29 s gelation time. These changes can be attributed to the imine crosslinking occurring between photo-generated aldehyde groups in capsules and hydrazide groups in HA macromolecules (Fig. 3C and D). Interestingly, the compressive strength of the hydrogel was also significantly improved because of the effective increasing of crosslinking density based on PLGA-NB capsules (Fig. 3E). The increase of the content of PLGA-NB capsules or pre-gelling polymers improved both final storage modulus and compressive modulus with nearly the same gelation time (Fig. 3C–E, and Supplementary Table 1). As a control, non-modified PLGA capsules did not significantly increase final storage modulus or compressive modulus compared to that of the HA-NB/HA-CDH hydrogels alone. We next evaluated the tissue adhesive strength of hydrogels via lap shear measurement based on ASTM F2255-05 standards. The tissue adhesive strengths of PLGA-NB capsule-loaded HA-NB/HA-CDH hydrogels were approximately 13.7 kPa (2.5 wt% solid content), and 23.4 kPa (5.0 wt% solid content), respectively, which were in the same order of magnitude as that of fibrin glue (13.73 ± 1.31 kPa), a commercialized tissue adhesive (Fig. 3F and Supplementary Table 1) used in surgery and wound care^35^.

Taken together, our results suggest that the multifunctional PLGA-NB capsules not only can encapsulate active cargo for timed pulsatile delivery, but also act as a crosslinker that strengthen and enhance the hydrogel network, making it a suitable platform for skin wound repair.

### PLGA-NB capsules suppress TGFβ signaling and enhance scarless wound repair in vivo

**Fig. 4:**
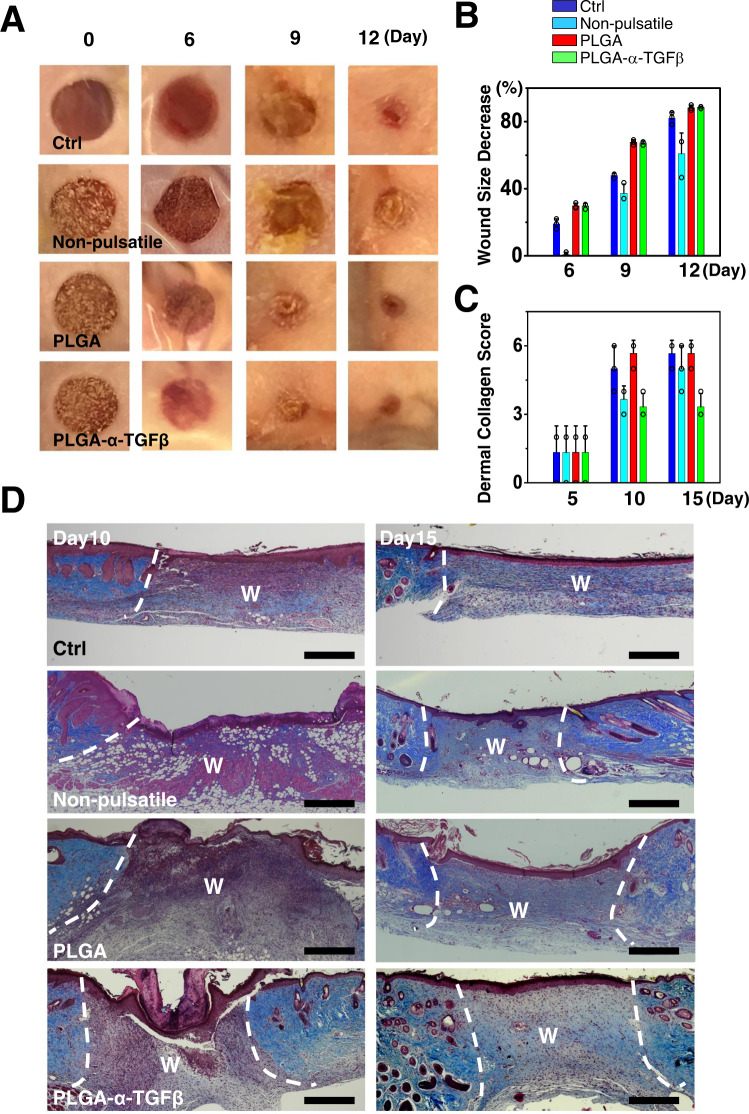
Delivery of TGFβ inhibitor with PLGA-NB capsules can enhance skin wound closure while suppressing scarring. **A**, **B** Representative wound images (**A**) and quantification of wound size (**B**) show that application of PLGA capsules with or without TGFβ inhibitor in HA-NB hydrogel can accelerate wound closure in murine skin with full thickness skin wounds. Delivery of TGFβ inhibitor in non-pulsatile release HA hydrogel inhibits wound healing. Treatment with alginate hydrogel was used as control (Ctrl). *n* = 3 independent samples. Data are presented as mean ± SD. **C** Quantification of collagen deposition and tissue fibrosis at different time points after wounding (Materials and Methods). *n* = 3 independent samples. Data are presented as mean ± SD. All error bars represent SD. **D** Trichrome staining of skin sections treated with PLGA capsules or PLGA capsules loaded with TGFβ inhibitor or control hydrogel at different time points post wounding. Dashed lines denote wound (W) boundary. scale bar = 500 μm. Source data are provided as a Source Data file.

We hypothesized that the PLGA microcapsules with timed release of TGFβ regulators would promote skin wound closure while suppressing scarring. To test this hypothesis, we prepared PLGA-NB microcapsules loaded with TGFβ1 inhibitor (SB-431542)^49^ and tested it with a murine skin wound repair model. To apply the material to skin wounds, 6% of loaded or unloaded PLGA-NB particles were mixed with HA-NB/HA-CDH hydrogel, because this ratio demonstrates the best tissue adhesive strength in vivo (Fig. 3F, and supplementary table 1). Full-thickness skin wounds were created on the dorsum of test animals (Fig. 4A). HA-NB/HA-CDH hydrogel with PLGA-NB capsules were introduced to the wounds and photogelation was initiated by UV illumination. Application of PLGA-NB capsules with or without SB-431542 (0.01 wt%) can significantly accelerate wound closure (Fig. 4A and B). By contrast, when SB-431542 was delivered by a traditional HA hydrogel in a non-pulsatile manner, skin wound healing was markedly inhibited (Fig. 4A and B).

**Fig. 5:**
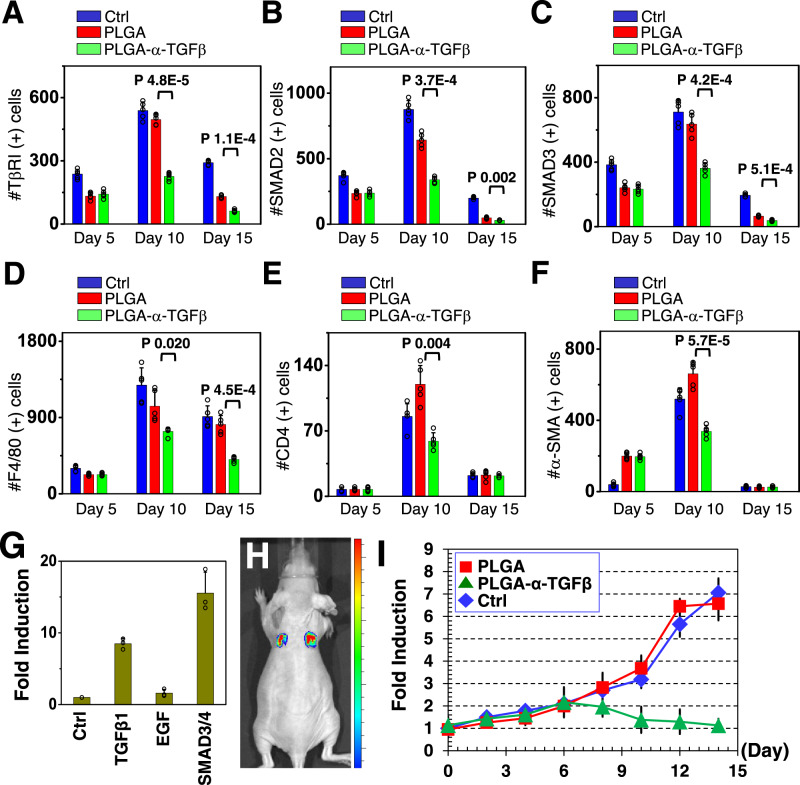
PLGA-NB delivery system can suppress TGFβ signaling after wound closure to inhibit skin scarring. **A-F** Quantification of TβRI (**A**), SMAD2 (**B**), SMAD3 (**C**), F4/80 (macrophage marker) (**D**), CD4 (**E**), and α-SMA (**F**) positive cells in skin after treatment of PLGA capsules loaded with TGFβ inhibitor. *n* = 5 independent samples. Data are presented as mean ± SD. *p* values for representative data are shown (One-tailed paired Student’s t-test). **G** Primary skin keratinocytes were transduced with vector encoding SBE-*luciferase* reporter. Bioluminescence activity was measured upon different stimulations. *n* = 3 independent samples. Data are presented as mean ± SD. **H** Intravital bioluminescent imaging of nude mice receiving skin grafts with expression of SBE-*luciferase* reporter. **I** Fold changes of bioluminescence signals after skin wounding upon treatment with PLGA capsules or PLGA capsules loaded with TGFβ inhibitor. *n* = 3 independent samples. Data are presented as mean ± SD. All error bars represent SD. Source data are provided as a Source Data file.

To evaluate the potential effects on scarring, we analyzed skin tissue samples 15 days post wounding. Trichrome staining indicated significantly less collagen deposition in wounds treated with inhibitor-loaded PLGA-NB capsules, but not with traditional HA hydrogel delivery system (Fig. 4C, D). The decrease of collagen deposition shows a dose-dependent response to the amount of inhibitor loaded to the PLGA-NB capsules (Supplementary Fig. 3A-B). Immunohistochemistry analysis further demonstrated diminished phosphorylation of TβRI, phospho-SMAD2, and phospho-SMAD3 in the TGFβ inhibitor treated group, consistent with the effect of TGFβ inhibition (Fig. 5A–C, and Supplementary Fig. 4). To assess potential changes in inflammatory responses during wound repair, we stained tissue sections with F4/80 for macrophages, and CD4 for helper T cells. Our results showed significantly reduced number of macrophages and CD4^+^ T cells in inhibitor-treated wounds (Fig. 5D, E, and Supplementary Fig. 4). Activated fibroblasts in the wounds were identified by staining with α-SMA (smooth muscle actin) (Fig. 5F, and Supplementary Fig. 4), which are also significantly decreased in the wounds treated with TGFβ inhibitor. Taken together, our results provide compelling evidence that PLGA-NB capsules with pulsatile release of TGFβ inhibitor can enhance skin wound repair while suppressing tissue fibrosis/scar formation in a murine model.

To directly determine the temporal specificity of the PLGA-NB-mediated drug release in vivo, we developed an intravital imaging approach to monitor the TGFβ signaling in live animals. To this end, we prepared a lentiviral vector encoding a *luciferase* reporter driven by the SBE (Smad-binding element) promoter^50,51^. Primary mouse skin keratinocytes infected with the virus exhibited greatly elevated bioluminescent signals upon co-transfection with Smad3/4 or stimulation with TGFβ, verifying the utility of the reporter in vitro (Fig. 5G). With our skin organoid and engraftment platform^52–54^, we established skin grafts with infected mouse epidermal progenitor cells and transplanted these grafts to nude host mice (Fig. 5H). Skin organoid transplants with SBE reporter were well taken by the animals, and grafted skin exhibited normal epidermal stratification and proliferation. Wound healing in skin grafts exhibits no significant difference as that in normal skin^54–56^ (Supplementary Fig. 5). When challenged by skin wounding, the skin transplants demonstrated a gradual increase in the bioluminescence, which peaked around 2 weeks post-wounding. Interestingly, while empty PLGA-NB capsules did not significantly alter the TGFβ signaling, application of capsules loaded with TGFβ inhibitor lead to dramatically reduced bioluminescent signals ~7 days post wounding (Fig. 5I). Our results provide compelling and direct evidence that the PLGA-NB capsules can lead to delayed and pulsatile release of the inhibitor in vivo to suppress skin scarring after initial skin wound closure and re-epithelialization.

### PLGA-NB capsules suppress scarring in the rabbit ear and porcine skin wounding model

Although murine skin provides a robust and rapid model for wound repair, wound healing in mouse skin does not lead to excessive scar formation. Mouse dorsal skin is loose, and wounds are healed by both skin contraction and rapid epithelial closure. Presence of abundant hair follicles in the mouse skin also accelerates re-epithelialization and reduces scarring upon wounding. In addition, the mouse dorsal skin lacks mechanical tension, which is critically important in tissue fibrosis. By contrast, it has been shown that wounds in rabbit ear skin can lead to reproducible and quantifiable formation of hypertrophic scars^57,58^. Compared with mouse dorsal skin, the rabbit ear skin is tight and less hairy. The skin wounds do not heal by contraction, and delayed re-epithelialization can lead to a raised scar, which resembles human hypertrophic scar.

**Fig. 6:**
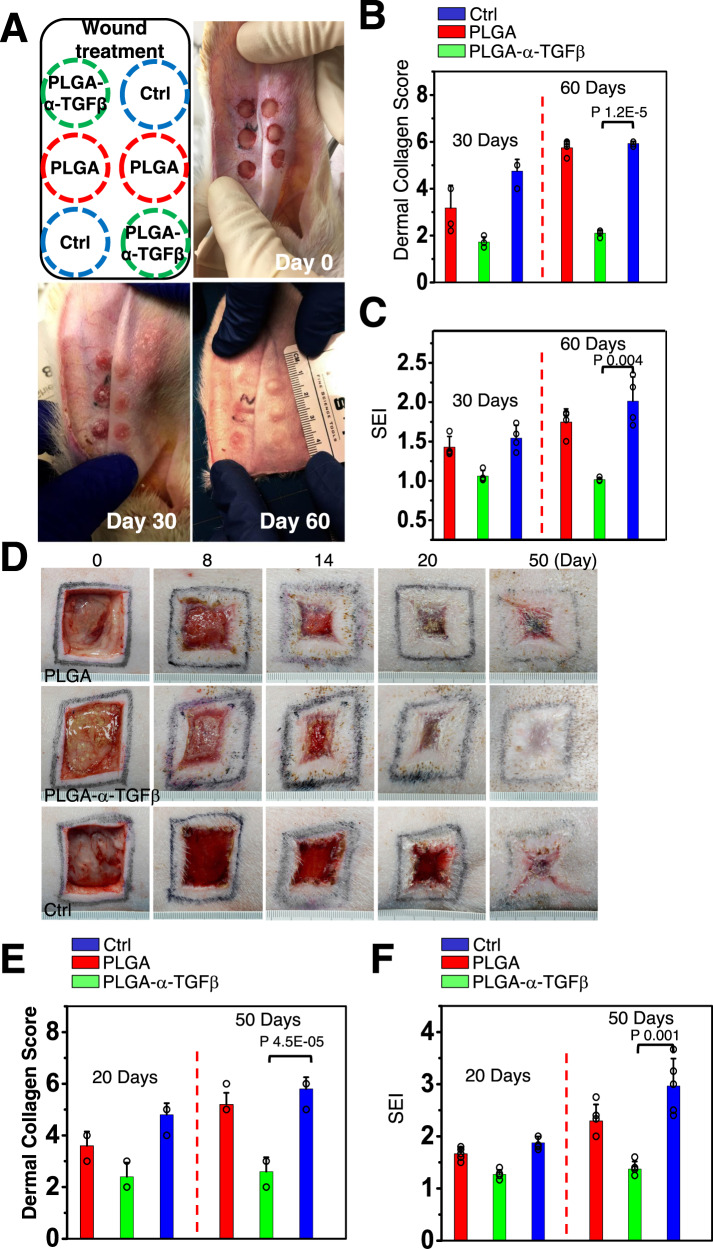
PLGA-NB delivery platform can promote scarless wound healing in rabbit and porcine skin wound healing model. **A** Representative images of rabbit ear skin wound healing and scar formation in differently treated groups. Left upper panel shows the respective treatment for each wound on the ear. **B**, **C** Quantification of collagen deposition and SEI (scar elevation index) at different time points after wounding. *n* = 4 independent samples. Data are presented as mean ± SD. All error bars represent SD. p values for representative data are shown (One-tailed paired Student’s t-test). **D** Representative images of porcine skin wound healing and scar formation in differently treated groups. **E**, **F** Quantification of collagen deposition and SEI (scar elevation index) at different time points after wounding. *n* = 5 independent samples. Data are presented as mean ± SD. *p* values for representative data are shown (One-tailed paired Student’s t-test). All error bars represent SD. Source data are provided as a Source Data file.

To test the potential effect of our material on this model, we created full-thickness wounds on the ventral side of the ears of adult rabbits (Fig. 6A). The untreated wounds usually healed within 15 days post-surgery. Application of the PLGA-NB capsules with or without TGFβ inhibitor by photogelation can promote wound closure as in mouse skin model (Supplementary Fig. 6A). Histological evaluation indicated significantly reduced cellularity and collagen fiber deposition in inhibitor treated wounds (Fig. 6B, and supplementary Fig. 6B). To assess skin scarring, we determined the scar elevation index (SEI) by histomorphometric analysis of skin tissue samples collected 30 days post wounding^57,58^. The SEI measures the ratio of total hypertrophied scar tissue area to the area of underlying dermis, providing an accurate and reproducible instrument to evaluate scar formation (Supplementary Fig. 6C). Ear wounds treated with TGFβ inhibitor via PLGA-NB microcapsules exhibited significantly reduced SEI compared with control wounds (Fig. 6C). The scar-suppressing effect of TGFβ inhibitor is long-term. Significantly reduced SEI and collagen deposition can be observed in inhibitor treated ear wounds 60 days after surgery (Fig. 6B and C).

Porcine skin resembles human skin anatomically and physiologically. The full-thickness excisional wounding model in porcine skin is one of the best preclinical wound healing models^59^. To determine the effectiveness of our platform in a preclinical setting, we examined healing and scarring of full-thickness cutaneous wounds on the dorsal skin of Yorkshire pigs. Consistent with the data from rodent skin models, application of the capsules with or without TGFβ inhibitors by photogelation can enhance wound closure (Fig. 6D, and Supplementary Fig. 6D). Healed skin wounds in TGFβ inhibitor treatment group had a flat surface without significant disfigurement and less scarring 50 days post-wounding. By contrast, wounds in the control groups exhibited nodularity and excessive wound distortion. Trichrome staining indicated significantly less collagen deposition and more mature collagen arrangement in wounds treated with inhibitor- loaded PLGA-NB capsules, characterized by well-formed and regularly aligned collagen bundles in the healed wounds (Fig. 6E and Supplementary Fig. 6E). Histological evaluation also indicated significantly reduced SEI for wounds treated with TGFβ-inhibitor containing capsules (Fig. 6F).

Together, our results strongly suggest that timed delivery of TGFβ inhibitor via PLGA-NB microcapsules in a photo-crosslinking hydrogel can significantly enhance wound closure while suppressing skin scarring in preclinical skin wounding and scarring models.

## Discussion

Hypertrophic scars often occur following deep trauma, severe burn injury, or surgical incision on human skin. Despite advances in skin wound management and improved survival rates, the incidence of hypertrophic scarring remains high^4–9^. Skin scarring can have a profound impact on the quality-of-life of patients, and pose a great challenge to the field. TGFβ signaling has been critically implicated in skin scarring, making them potential targets for treatment^7,14–16^. However, as the role of TGFβ signaling in cutaneous wound repair is multifaceted, the inhibition of TGFβ activity has to be specific to the resolution/tissue remodeling phase of wound healing in order to suppress scarring^60^. In this study, by integrating advanced material synthesis with molecular and cellular studies of skin wound repair, we were able to design a PLGA-based capsule platform with specific composition and degradation kinetics. Application of this system in vivo can deliver TGFβ signaling inhibitors in a spatiotemporal specific manner and achieve scarless wound repair in both rodent and porcine skin wounding models.

Material-bio interactions play a key role in skin wound healing. The interface comprises the dynamic physicochemical interactions, kinetics and thermodynamic exchange between synthetic material surfaces and the surfaces of biological components (e.g., proteins, membranes, phospholipids, and biological fluids). In skin, the material-tissue integration can provide stable biological fixation, reduce the risk of infection, and enhance the wound healing process^61,62^. Although various material including hydrogels have been tested for skin wound repair, it remains challenging to integrate synthetic material with surrounding tissues because there are limited surgical methods and adhesives that can be used for bonding or fixing the material^61–64^. In this study, we employed a photo-inducible imine crosslinking reaction based hydrogel for skin wound treatment^34–36^. This unique photo-gelation method can effectively crosslink a wide variety of biocompatible macromolecules with the PLGA-NB capsules without chemical modification. The crosslinking can lead to successful integration of the material with an intimate and chemically bonded interface between the microcapsules, hydrogel and wound surface, promoting the healing of skin wounds.

Skin wound healing is a dynamic and interactive process, which is usually divided into four different stages: hemostasis, inflammation, re-epithelization, and resolution/tissue remodeling^1–3^. Although spatiotemporal overlap may exist, key time windows can be identified for each individual stage. PLGA-NB capsules can be prepared with different composition and fabrication to tailor cargo release kinetics. Thus, it is conceivable that more advanced wound dressing material can be prepared with PLGA-NB capsules, which can release different factors at different target time windows. For instance, capsules with faster release kinetics can be used to deliver a variety of growth factors, including TGFβ that can promote keratinocyte proliferation, granulation tissue formation, and angiogenesis to enhance skin wound repair. Beyond traditional wound treatment, it has also been well-documented that autologous or allogenic epidermal progenitor cells can be used for treatment of massive burn wounds and chronic skin lesions, such as diabetic foot ulcers^2^. With its strong biocompatibility, our material will allow and support potential transplantation of CEA (cultured epidermal autograft) device or allogenic skin grafts. In addition, the unique photogelation process of our material can also be used to facilitate skin wound healing by stem cell spray^2^.

In closing, fabrication of capsules with timed pulsatile release of cargo represents a significant advancement toward scarless wound repair, which can be also applied to other aspects of tissue engineering and regenerative medicine.

## Methods

### Reagents

Hyaluronic acid (HA; MW: 48 or 340 kDa), Carboxylic poly(lactic-co-glycolic acid) (OH-PLGA-COOH; MW: 50 kDa, LA:GA = 75:25) were purchased from Sigma-Aldrich, TGF-β inhibitor SB431542 is purchased from Selleckchem, Inc. Polyvinyl alcohol (PVA; MW: 95,000) and poly(ethylene glycol) (PEG; MW 400) were obtained from Sigma-Aldrich, Inc. Milli-Q185 water (Waters, Saint-Quentinen-Yveline, France) was used in all experiments. The fluorescent dyes, 1,1′-dioctadecyl-3,3,3′,3′-tetramethylindocarbocyanine perchlorate (DiI) and 1,1′-dioctadecyl-3,3,3′,3′-tetramethylindodicarbocyanine perchlorate (DiD), were obtained from Fisher Scientific, Inc. Anti-phospho-TGFβR1(P-S165) was from Aviva Systems Biology (OAAI00768, polyclonal, 1:200 dilution). Anti-phospho-SMAD2 (S465, S467) and anti-phospho-SMAD3 (S423, S425) were from Invitrogen (44-244 G and 44-246 G, polyclonal, 1:100 dilution for both). Anti-alpha-smooth muscle actin was from DAKO (M0851, clone 1A4, 1:100 dilution). Anti-F4/80 was from AbD Serotec (MCA497GA, clone Cl:A3-1, 1:200 dilution). Anti-CD4 + was from Affymetric eBioscience (14-9766-82, clone 4SM95, 1:600 dilution). All other chemicals were of analytical grade and obtained from Sigma-Aldrich, Inc.

### Synthesis of pre-gelling polymers

The synthesis of NB-modified poly(lactic-co-glycolic acid) (PLGA-NB): NB was carried out essentially as following^36^. Carboxylic poly(lactic-co-glycolic acid) (5 g, molecular weight 50 kDa) was dissolved in dichloromethane, and 1-ethyl-3-(3-dimethylaminopropyl)carbodiimide hydrochloride (39 mg, 0.2 mmol, EDC·HCl), N-hydroxysuccinimide (23 mg, 0.2 mmol, NHS) in solid form were added. Then, NB (65 mg, 0.2 mmol) was dissolved in dimethyl sulfoxide (DMSO) and added into the above solution to stir overnight in the dark. After the reaction, the solvent was evaporated in vacuum, and the mixture was reprecipitated using ethyl alcohol three times. The crude product was centrifuged and then dried at 50 °C in vacuum for 12 h to remove the residual ethyl alcohol. ^1^H NMR analysis was performed to determine the substitution degree of the nitrobenzyl group (98.5%). TopSpin software was used for ^1^H NMR Data collection.

The synthesis of NB-modified hyaluronic acid (HA-NB): HA (2 g, molecular weight 340 kDa) was dissolved in 100 mL 0.1 M MES solution (pH = 5.17), and 4-(4,6-Dimethoxy-1,3,5-triazin-2-yl)-4-methyl morpholinium chloride (0.4 g, 1.36 mmol, DMTMM) was added. Then, NB (60 mg, 0.18 mmol) was dissolved in dimethyl sulfoxide (DMSO) and added into the above solution to stir overnight in the dark at 30 °C. After the reaction, the solution was dialyzed against deionized water for 3 days followed by freezing and lyophilization. ^1^H NMR analysis was performed to determine the substitution degree of nitrobenzyl group (3.5%).

### The synthesis of carbohydrazide-modified hyaluronic acid (HA-CDH)

HA (2 g, molecular weight 48 kDa) was dissolved in 50 mL of deionized water, and 1-ethyl-3-(3-dimethylaminopropyl)carbodiimide hydrochloride (0.2 g, 1 mmol, EDC·HCl), N-hydroxysuccinimide (0.12 g, 1 mmol, NHS) in solid form were added. Carbohydrazide (0.09 g, 1 mmol) was dissolved in deionized water and added into the above solution to stir overnight. The solution was then dialyzed against deionized water for 3 days followed by freezing and lyophilization. The substitution degree of hydrazide modifications (10.8%) was determined following literature procedure using trinitrobenzene sulfonic acid (TNBS) assay, which gives a quantitative assessment of free carbohydrazide residues using UV spectroscopy^36^.

### Timed release capsule fabrication

All capsules were produced by W/O/W emulsion method followed by solvent evaporation technique. We first prepared 50 μL deionized water with 2.5 mg PEG400 to get the inner water phase. After 15 min of sonication, this solution was droplet added into a solution of 100 mg PLGA-NB or PLGA dissolved in 1 mL of chloroform with vigorously stirring. This process was performed over at least 10 min to allow for emulsification. The resulting water-in-oil (W/O) emulsion was then transferred into a 15 mL aqueous solution composed of 1% (wt/vol) PVA used as an emulsion stabilizer. The double emulsion (W/O/W) was obtained using 3000 rpm stirring with a magnetic stirrer (C-MAG HS 7; IKA Works, Inc.), and then placed in a laboratory fume hood for 6 h for solvent evaporation. The capsules with a blank core recovered after centrifugation were washed with deionized water and then lyophilized with the freeze dryer (Virtis Benchtop; SP Industries, Inc.).

Capsules with timed release TGF-β inhibitor were produced by the same method, except during the preparation of the inner water phase, when different weight ratio of TGF-β inhibitor was added to the deionized water and PEG400. Similarly, to create the bovine serum albumin (BSA)-loaded capsules, 5 mg BSA was dispersed in deionized water and PEG400 when generating the inner water phase. Likewise, for the timed release capsules containing FRET fluorophores, DiI and DiD (2.5 nmol of DiI; DiD:DiI 4:1) were dissolved in ionized water and PEG400 for the inner water phase.

Capsule size, surface morphology and inner hollow structure were determined by light microscopy (EVOS FL; Advanced Microscopy Group), high-resolution Field-Emission Scanning Electron Microscopy (FESEM; Merlin; Carl Zeiss, Inc.) and Inverted Fluorescence Microscopy (Nikon eclipse). SmartSEM software was used for FESEM data collection. Ti2 Control Ver1.0.2 software was used for Inverted Fluorescence Microscopy data collection. For light and fluorescence microscopy, the capsules were lyophilized and re-dispersed onto a glass slide prior to imaging. For fluorescence imaging, the wavelengths of the blue channel were 340–380 nm (excitation) and 435–485 nm (emission); the wavelengths of the red channel were 528–553 nm (excitation) and 590–650 nm (emission). For FESEM, the dispersed PLGA-NB capsule in water was dropped onto a silica chip and air dried. The silica chip with PLGA-NB capsules was coated with platinum at 20 mA for 70 s under vacuum. The images were taken using FESEM at an acceleration voltage of 2 kV.

For control alginate microparticles, it was produced as following^36^. Briefly, 10 ml of alginate solution (2.0 wt %) containing FRET fluorophores, DiI and DiD (5 nmol of DiI; DiD:DiI 4:1) or BSA (10 mg) was introduced into a large beaker containing 200 mL of CaCl_2_ solution (1 mol/L) by a pump to be directly solidified. The microparticles were then separated by centrifugation after solidification and washed twice with physiological saline to remove the residual CaCl_2_ on the surface.

### Cytotoxicity evaluation of the capsules

The Cytotoxicity of human colorectal carcinoma (Caco-2) cells and human epithelial carcinoma (HeLa) cells was assessed by the Cell Counting Kit-8 (CCK-8) method. Caco-2 cells and HeLa cells were obtained from ATCC. This colorimetric cell proliferation kit allows for easy and reliable colorimetric determination of viable cell numbers with excellent sensitivity and linearity. The cells were maintained according to routine cell culture procedures. To determine cell viability after exposure of all cell lines to different concentrations incubated for 24 h, the CCK-8 assay was performed according to manufacturer’s instructions. Briefly, the cells were seeded in 96-well plates at 37 °C in humidified atmosphere (90% humidity), 7.5% CO_2_, Dulbecco’s minimal essential medium, 1% (wt/vol) nonessential amino acids, 1% (wt/vol) glutamine, 10% (vol/vol) fetal bovine serum, penicillin, (100 U/mL), and streptomycin (100 μg/mL). When 50% confluence was reached, the tested capsules were dispersed in cell growth medium at 1, 10, and 100 μg/mL and added to the wells. After incubating for 24 h, the medium was removed, and after washing the cells with PBS, CCK-8 reagent was added to each well followed by 2 h of incubation at 37 °C. The absorbance was measured using a microplate reader (Synergy Neo; BioTek Instruments, Inc.) at 450 nm. Gen5 software was used for microplate reader data collection.

### Characterization of capsule-loaded hydrogels

The pre-gelling polymers of HA-NB, HA-CDH, PLGA, or PLGA-NB were mixed according to different requirements in Dulbecco’s Phosphate Buffer Saline (D-PBS, pH 7.4) at 37 °C. Then the above pre-gelling solution was irradiated (365-nm LED, 20 mW/cm^2^) and then subjected to different measurements.

Dynamic rheology experiments were performed on HAAKE MARS III photorheometer with parallel-plate (P20 TiL, 20-mm diameter) geometry and OmniCure Series 2000 (365 nm, 20 mW/cm^2^) at 37 °C. RheoWin software was used for rheometer data collection. Time sweep oscillatory tests were performed at a 10% strain (CD mode), 1 Hz frequency and a 0.5 mm gap for 300 s. Strain sweeps were performed to verify the linear response. The gelation time was determined as the time when the storage modulus (*G*′) surpassed the loss modulus (*G*″). The final storage modulus was determined as the point when the storage modulus (*G*′) reached complete gelation.

Mechanical tests were carried out on as-prepared hydrogels using GT-TCS-2000 universal material testing machine with a capacity of 100 N. Gotech materials testing software was used for mechanical tests data collection. For compression tests, hydrogel samples were prepared to have cylindrical shape with 10-mm diameter and 3-mm length and the speed was set at 1 mm/min. The hydrogels after complete gelation under light irradiation (365-nm LED, 20 mW/cm^2^) were subjected to compression tests.

Tissue adhesive strength test was performed as following^36^. Briefly, a 3.5 × 2.5 cm fresh hog casing was attached to an 8 × 2.5 cm glass slide by cyanoacrylate glue. Then, 100 μL of gel precursor was uniformly dispersed on the surface of the hog casing. A second hog casing was attached to a glass slide and placed on the first glass slide. The obtained test samples were irradiated (365-nm LED, 20 mW/cm^2^) to allow the gel precursor to gel in situ. One side of the sample was fixed, and increasing pull strength was applied to the other side by GT-TCS-2000 universal material testing machine. Then, the tissue adhesive strength of the hydrogel was calculated according to the below equation. Four samples were tested to determine the adhesive strength of each group (*n* = 4).

$$Tissue\;adhesive\;strength = F/A$$

*F*: pulling stress; *A*: adhesive area

### In vitro release kinetics of timed release capsule

BSA loaded capsules were placed into 5 mL phosphate-buffered saline (PBS) in a centrifuge tube and incubated on a shaker at 37 °C. Release was then measured everyday using a Pierce™ BCA Protein Assay Kit (Thermo Fisher Scientific Inc.). Results were quantified using a standard curve and normalized to total cumulative release (*n* = 5).

### In vivo release kinetic of timed release capsule

All mice used in this study were bred and maintained at the ARC (animal resource center) of the University of Chicago in accordance with institutional guidelines. All the experimental procedures on live animals (mouse, rabbit, and pig) were carried out in line with the Institutional Animal Care and Use Committee (IACUC) approved protocols of the Animal Care Center at the University of Chicago and Changhai Hospital, Shanghai, China. WT CD1 mice were obtained from the Transgenic Core Facility at University of Chicago. All the mice were housed under pathogen-free conditions in the ARC (Animal Resources Center) at the University of Chicago under a 12 h light-dark cycle. Housing facility maintains a temperature at 70–73° (average 72) and humidity at 40–50% (average 44%). All the subjects were not involved in any previous procedures. Capsules were sterilized prior to surgery using a 20 µl drop of 70% ethanol. Before injection, mice were anesthetized using continuous inhalation of 3% isoflurane and had their injection site sterilized with ethanol. 30 mg of capsules were injected subcutaneously into the dorsum of each animal. Mice were imaged using an in vivo imaging system (IVIS 200; Xenogen Corporation) daily. Living Image® software was used for in vivo imaging system. Fluorescent images were then collected using 560/620 nm excitation/emission filter sets with an 1.00 s exposure time, F-Stop setting of 1, medium binning and subject height of 1.5 cm. Cumulative release was assigned to the maximum and minimum overall fluorescence in the region of interest to match particular capsules’ release and background signal, respectively.

### Mouse skin wound model and treatment

*CD1* male mice aged 6–8 weeks were used in this study. The mice were anesthetized using continuous inhalation of 3% isoflurane. After shaving, 6 wounds were created on the dorsal side of each mouse by removing full-thickness skin via 6-mm punch biopsy. After application of hydrogel and capsules, the wounds were illuminated with LED light (365 nm LED, 20 mW/cm) for 3 min to activate the crosslinking reaction. Tegaderm^TM^ films (3 M Inc.) were used to cover the wounds and prevent water loss in all the mice until the wounds are fully epithelialized. Serving as a control, we have used 2.5 wt% HA hydrogel to deliver the TGFβ inhibitor as a traditional non-pulsatile delivery platform (Fig. 4B and C).

At day 5, 10 and 15 post-surgery, 3 mice in each group were euthanized and the wounded skin removed, fixed in formalin, embedded in paraffin, and sectioned. Hematoxylin and eosin (H&E), trichrome, phopho-TβRI antibody, phospho-SMAD2 antibody, phospho-SMAD3 antibody, F4/80 antibody, anti-alpha smooth muscle actin antibody (α-SMA), and CD4^+^ antibody staining was used for histological observations. Antibodies were diluted according to manufacturer’s instruction, unless indicated otherwise. The sections with phopho-TβRI, phospho-SMAD2, phospho-SMAD3, F4/80, anti-alpha smooth muscle actin (α-SMA) and CD4^+^ staining were observed under the microscope (Eclipse Ti2; Nikon Inc.) at 400× magnification. Five fields were randomly selected from each section to count the phospho-TβRI, phospho-SMAD2, phospho-SMAD3 positive stromal cell, F4/80 positive macrophages, α-SMA positive fibroblast cell, and CD4^+^ positive T-cells. Immunoreactive cells were quantified as the mean cell count expressing the appropriate positive marker per high-power field (HPF). The severity of dermal fibrosis (dermal collagen score) was scored on a 0-6 scale based on trichrome stain by combining the area (0-3, 0: no stain, 1: staining present in 1/3 of the whole dermal thickness, 2: 1/3–2/3 of the dermal thickness, 3: more than 2/3 of the dermal thickness) and the intensity (0–3, 0: no staining, 1: weak staining, 2: intermediate staining, 3: strong staining). The values were measured twice by two blinded examiners and then averaged. Histological data are expressed as mean ± standard deviation (SD). Statistical analysis was performed by Student’s t-test. A *p* value of < 0.05 was considered significant.

### Skin organoid culture and transplantation

Skin grafting was carried out on nude female mice aged 6–8 weeks^53,65^. Decelluralized dermis (circular shape with 1 cm diameter) was prepared by EDTA treatment of newborn *CD1* mouse skin^66^. A total of 1.5 × 10^6^ cultured keratinocytes were seeded onto the dermis in cell culture insert. After overnight attachment, the skin culture was exposed to the air/liquid interface. For grafting with skin organoids, nude mice were anesthetized. One or two split-thickness wounds were created on the dorsal skin (~1 cm^2^ each). Skin organoids with dermal matrix as supporting material were transferred to the wounds and sealed with veterinary surgical glue. The grafts were wrapped with non-adhesive gauze and surgical gauze. About one week, the wound dressing was removed to expose the graft to air. Skin organoids become incorporated into the adjacent nude skin 2 weeks after the surgery, and are stably grafted for more than 6 months. The grafts resembles normal nude skin in histology, and have similar wound healing response as normal skin^54–56^. To examine wound response in skin grafts, 3 mm or 6 mm full-thickness punch wounds were created inside the graft, and wound healing response was monitored as described above.

### Rabbit ear hypertrophic scar model and treatment

We utilized a reproducible and quantifiable dermal ulcer model established by Mustoe et al.^67^. Adult *New Zealand* White female rabbits were used for this model. The rabbits were anesthetized with an intramuscular injection using ketamine (60 mg/kg) and xylazine (5 mg/kg). Wounds were created on the ventral side of the ears using a 7-mm dermal biopsy punch to reach the cartilage. The cartilage was meticulously notched without full dissection, while epidermis, dermis, and perichondrium were scrupulously removed using a dissecting microscope. This process would delay epithelialization and increase the degree of hypertrophic scaring, which leads to persistent scar elevation^58^. After hydrogel and capsules were applied, the wounds were illuminated (365 nm LED light, 20 mW/cm) for 3 min to activate crosslinking. Tegaderm^TM^ films were applied to cover the wounds until full epithelialization of the wounds. The wounded skin tissues were collected after the experiments. Samples were resected, fixed in formalin, and embedded in paraffin. Sections were made across the most elevated portion of the scar and stained with H&E and trichrome. For histomorphometric analysis, the scar elevation index (SEI) which measures the ratio of total scar connective tissue area to the area of underlying dermis is assessed^58^. The thickness of the dermis is determined based on the adjacent unwounded dermis. Histological data are expressed as mean ± standard deviation (SD). Statistical analysis was performed by a paired two-tailed Student’s t-test. A *p* value of < 0.05 was considered significant.

### Porcine skin wound healing model

Three male *Yorkshire* porcine (25–30 kg) were fasted for 12 h before surgery. Briefly, the animal was anesthetized with an injection of ketamine (20 mg/kg, IM), followed by propofol (1 mg/kg, IV), and then intratracheally intubated and ventilated. Anesthesia was maintained with 4 mg kg^−1^ h^−1^ propofol during the surgery. The anesthetized pigs had their backs depilated, immobilized, and placed in a dorsal position. The dorsal skin was cleaned with water and soap, and sterilized with iodine and 75% alcohol. For the creation of the defect, 12 areas of skin wound were created by removing 3 cm × 3 cm of full thickness skin in the central back along the thoracic and lumbar area. Incisions were with a surgical blade to the panniculus carnosus layer and the overlying skin was excised. After application of hydrogel and capsules, the wounds were illuminated with a 365 nm LED light (20 mW/cm) for 3 min to activate crosslinking. Large Tegaderm bandages (3 M Inc.) were used to cover the wounds and followed by 3 M loban 2 antimicrobial drape around the perimeter, forming a watertight dressing, and finally a specially designed jacket to hold the bandage in place. The wounded skin tissues were collected at day 20 and 50 post-surgery. Samples were resected, fixed in formalin, and embedded in paraffin, followed by H&E and trichrome staining for histomorphometric observations.

### Statistics and reproducibility

FESEM, phase-contrast imaging, epifluorescence imaging, and immunological staining experiments have been replicated three times independently to ensure reproducibility of results.

### Reporting summary

Further information on research design is available in the Nature Research Reporting Summary linked to this article.

## Supplementary information

Supplementary Information

Reporting Summary

## Data Availability

For this study, we will make our data available to the scientific community, which will avoid unintentional duplication of research. All the research data will be shared openly and in a timely manner in accordance with the most recent NIH guidelines (http://grants.nih.gov/grants/policy/data_sharing/). All data supporting the findings from this study will be available from the corresponding author upon reasonable request. Source data are provided with this paper.

## Source data

Source Data
