# Supplementary material for: A pulsatile release platform based on photo-induced imine-crosslinking hydrogel promotes scarless wound healing
Source: Nat Commun. 2021 Mar 15;12:1670. doi: 10.1038/s41467-021-21964-0 (PMC7960722; doi:10.1038/s41467-021-21964-0)
Supplement: Supplementary file 1 — Supplementary Information [file 41467_2021_21964_MOESM1_ESM.pdf]

SUPPLEMENTARY INFORMATION:

Supplementary Table 1

Mechanical property and characteristics of HA-NB hydrogel with PLGA-NB microcapsules.

	Component	Gelation Time (s)	Final Storage Modulus (Pa)	Compressive Modulus (kPa)	Adhesive Strength (kPa)
1	2.5% wt HA-NB/HA-CDH	27±2	268±12	14.6±2.6	11.3±1.2
2	2.5% wt HA-NB/HA-CDH + 3% wt PLGA-NB	28±2	307±23	15.4±3.2	12.3±0.6
3	2.5% wt HA-NB/HA-CDH + 6% wt PLGA-NB	29±2	353±31	19.1±2.3	13.7±1.7
4	2.5% wt HA-NB/HA-CDH + 9% wt PLGA-NB	30±3	412±28	26.6±3.7	12.1±0.9
5	2.5% wt HA-NB/HA-CDH + 6% wt PLGA	28±2	285±16	17.3±2.1	11.6±1.0
6	5% wt HA-NB/HA-CDH	12±1	1543±87	64.5±4.6	21.3±2.1
7	5% wt HA-NB/HA-CDH + 6% wt PLGA-NB	14±2	1987±104	70.4±5.4	23.4±3.2

Supplementary Figure 1

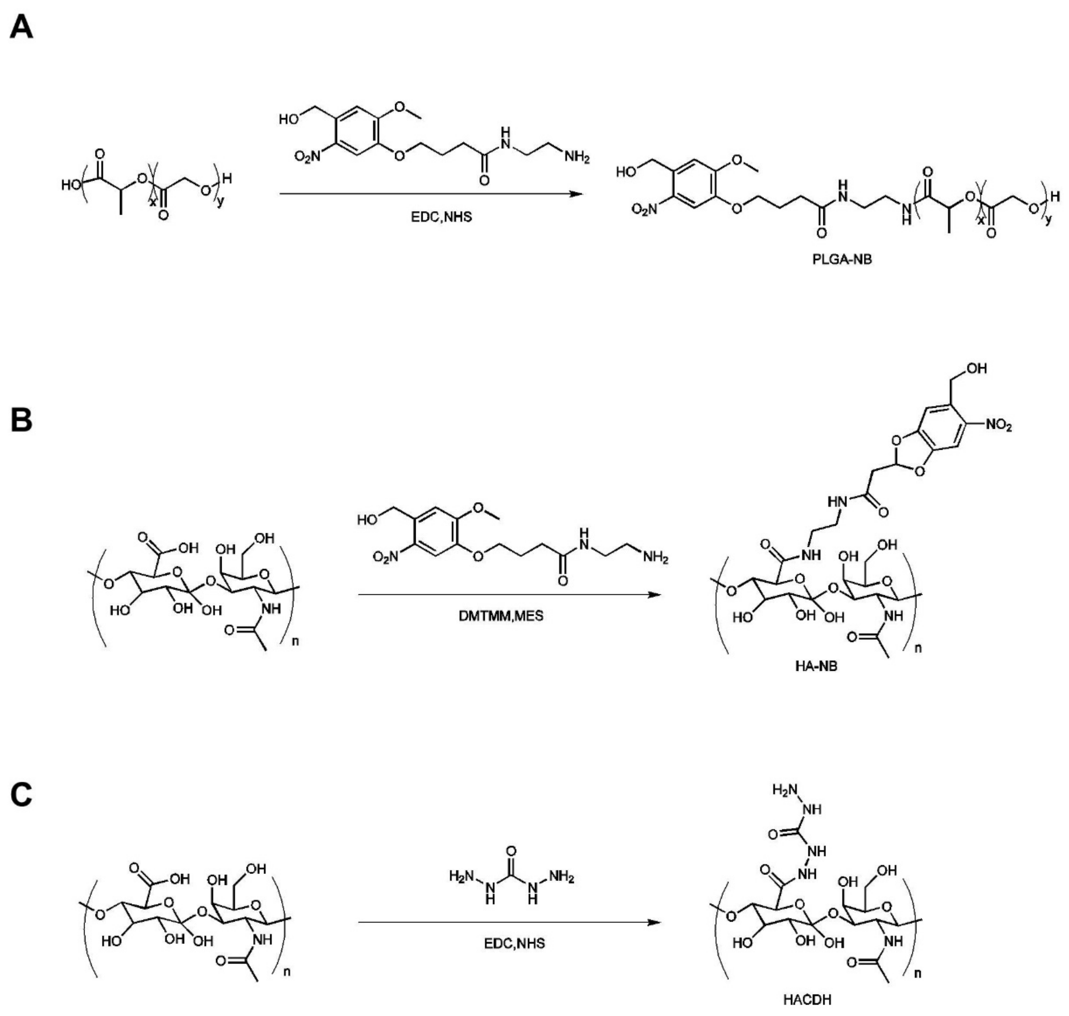

Supplementary Figure 1. Schematic illustration of the synthesis of the pre-gelling polymers: PLGA-NB (A); HA-NB (B); and HA-CDH (C).

Supplementary Figure 2

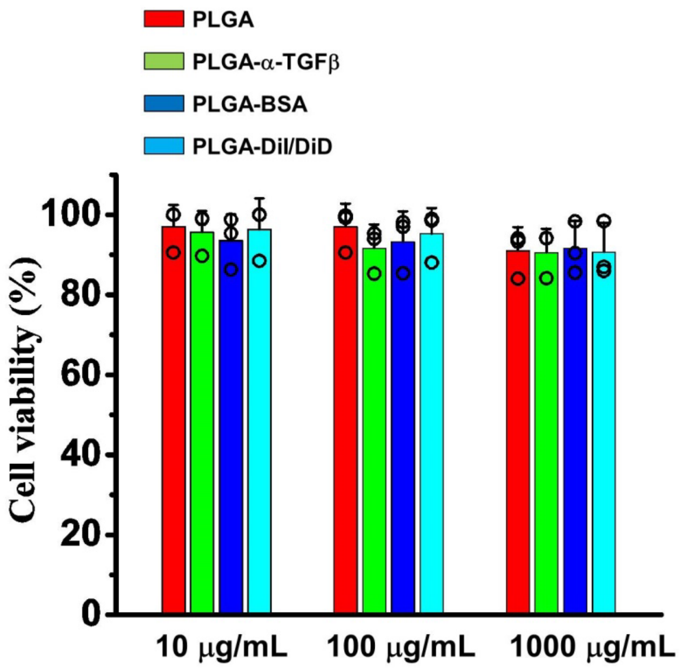

Supplementary Figure 2. Quantification of cell viability *in vitro* for empty PLGA-NB particles or particles loaded with different cargoes. n=3 independent samples. All error bars represent SD. Source data are provided as a Source Data file.

Supplementary Figure 3

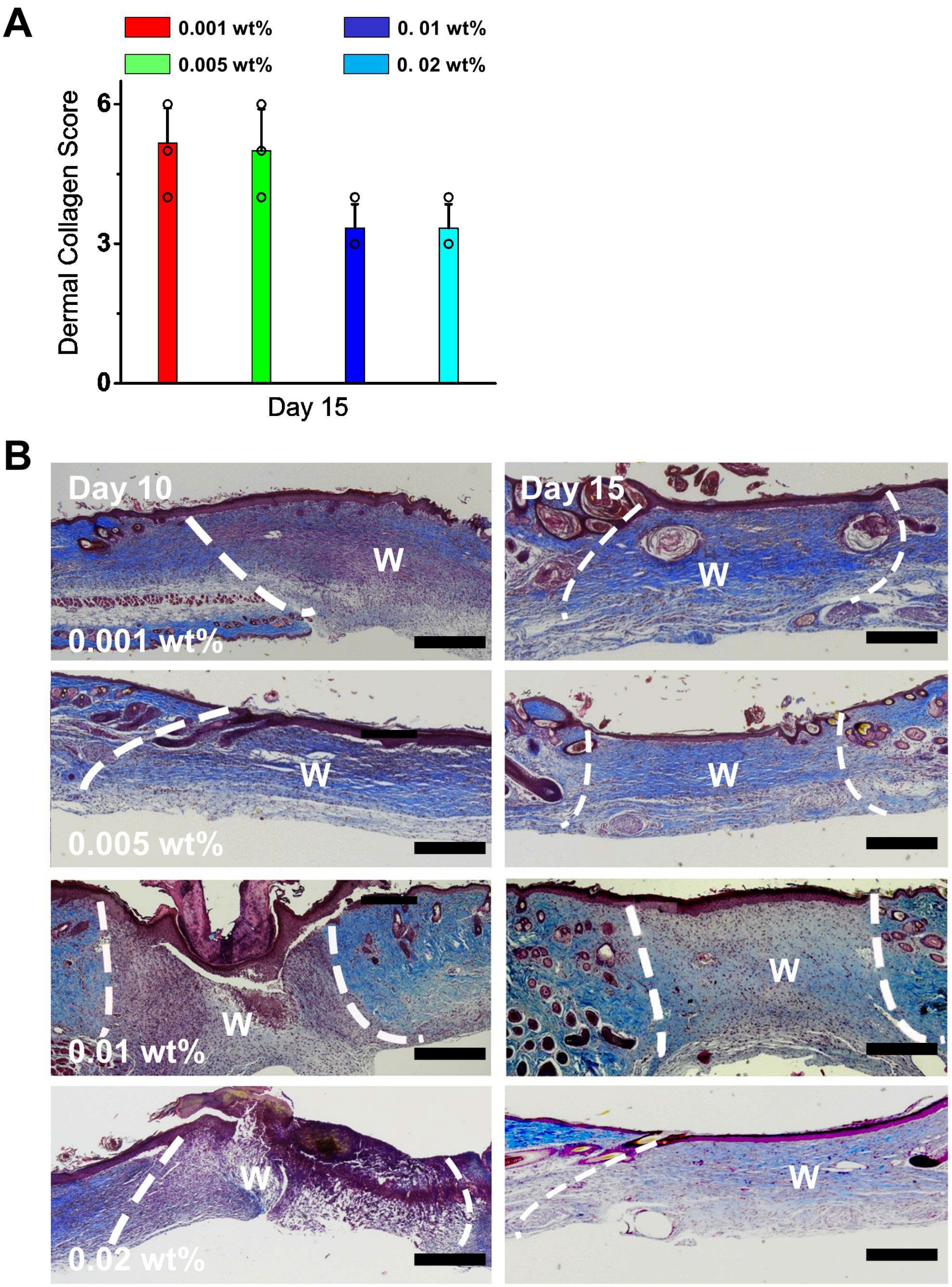

Supplementary Figure 3. (A) Quantification of collagen deposition and tissue fibrosis in healed wounds after TGFβ inhibitor treatment (in PLGA-NB capsules) at different dosages (0.001 wt% - 0.02 wt%). n=6 independent samples. All error bars represent SD. (B) Trichrome staining of skin sections treated with TGFβ inhibitor at different dosages. Dashed lines denote wound (W) boundary. Scale bar = 500 μm. Source data are provided as a Source Data file.

Supplementary Figure 4

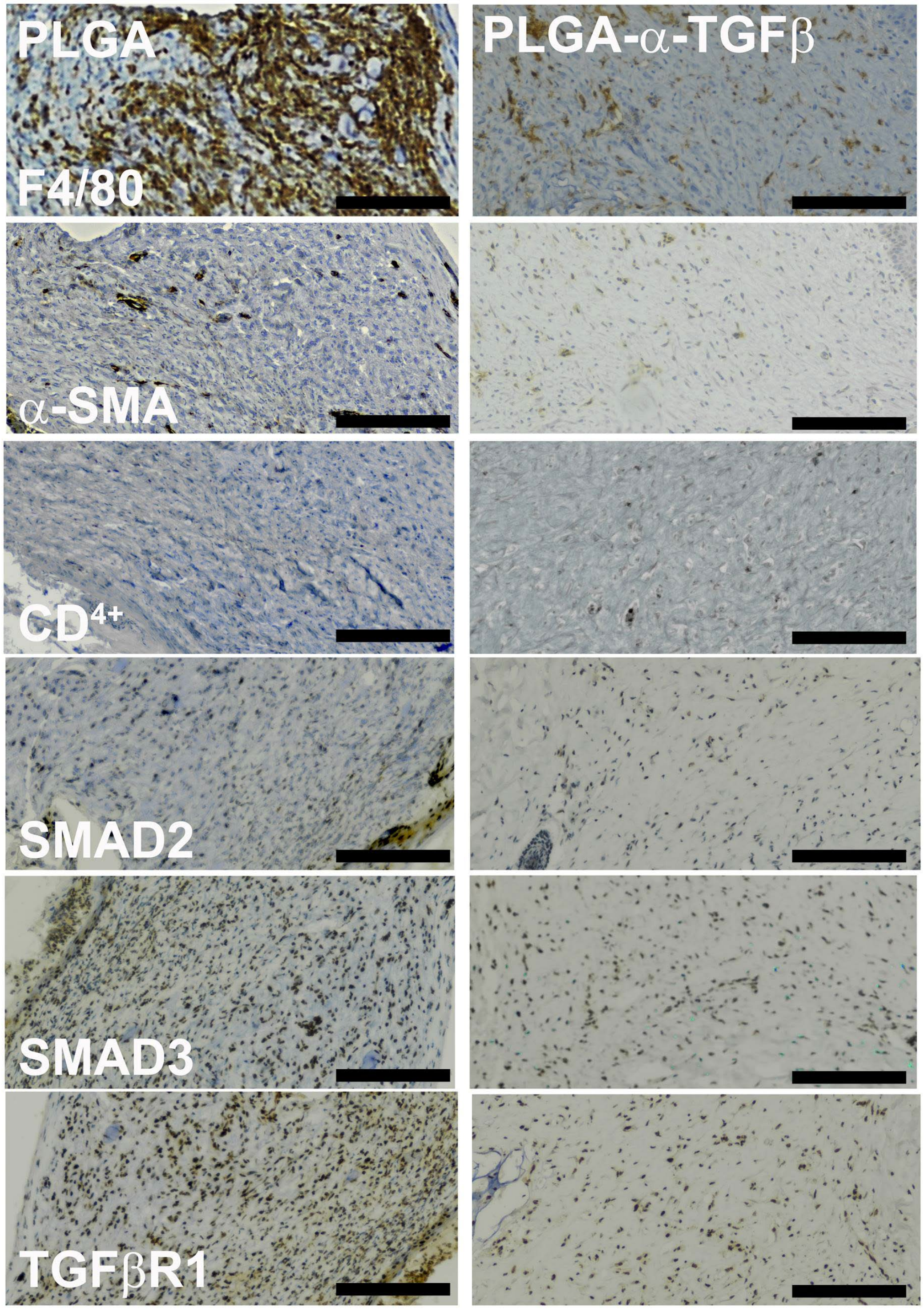

Supplementary Figure 4. Tissue sections were collected mouse skin wounds treated with control (Ctrl) or PLGA-NB capsules loaded with TGFβ inhibitors at 15 days post-wounding. Sections were stained with different antibodies to visualize TGFβ signaling and tissue inflammatory responses as indicated. Scale bar = 200 μm.

Supplementary Figure 5

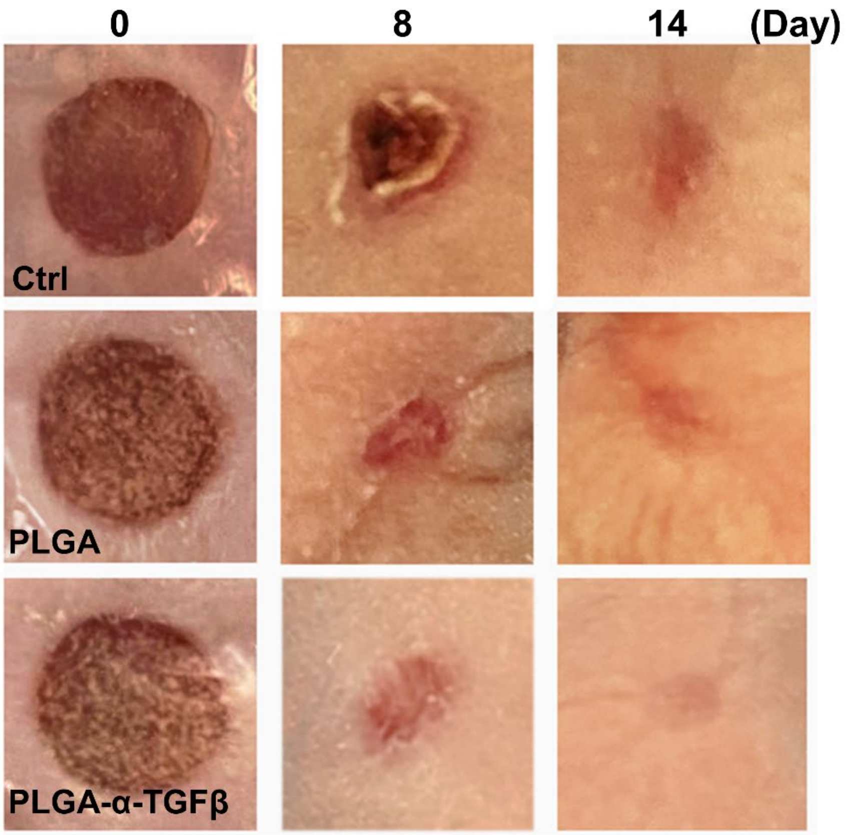

Supplementary Figure 5. Representative images of wounds in skin grafts at different time points. The wounds in skin grafts were treated with PLGA capsules with or without TGFβ inhibitor in HA-NB hydrogel, or alginate hydrogel as control (Ctrl), as shown in Figure 4A.

Supplementary Figure 6

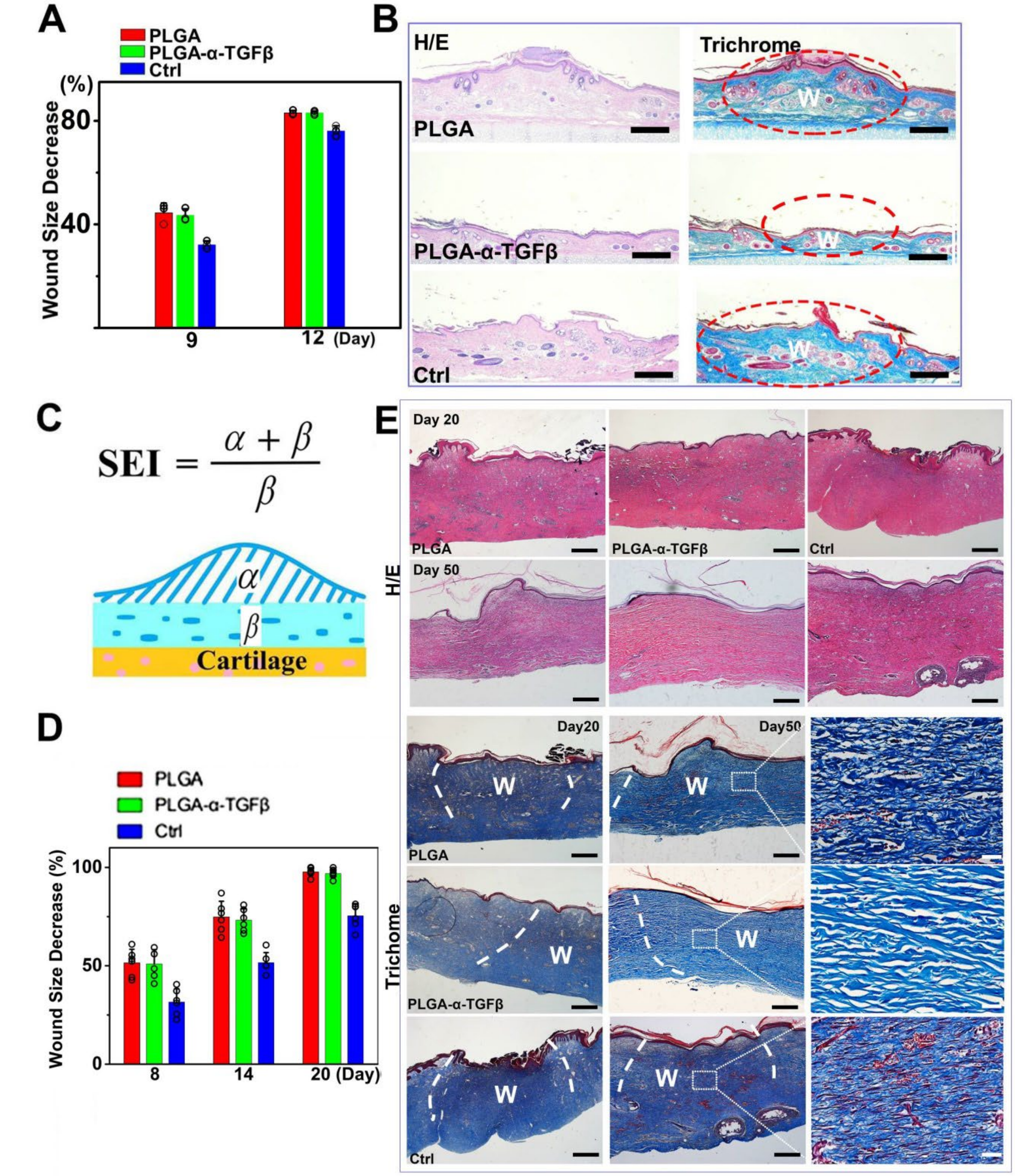

Supplementary Figure 6. Delivery of TGFβ inhibitor with PLGA-NB capsules can enhance skin wound closure while suppressing scarring. (A) Quantification of skin wound closure in rabbit ear

wounds after treatment with PLGA or PLGA particles loaded with TGFβ inhibitor. n=3 independent samples. Error bars represent SD. (B) H/E and trichrome staining of rabbit ear skin sections treated with PLGA capsules or PLGA capsules loaded with TGFβ inhibitor. Scale bar = 500 μm. (C) Schematic illustration of SEI (scar elevation index) measurement and calculation. (D) Quantification of skin wound closure in porcine skin wounds after treatment with PLGA or PLGA capsules loaded with TGFβ inhibitor. n=6 independent samples. Error bars represent SD. (E) H/E and trichrome staining of porcine skin sections treated with PLGA capsules or PLGA capsules loaded with TGFβ inhibitor. Scale bar at Day 20, Day 50, Enlarged figure = 500 μm, 200 μm, 40 μm, respectively. Source data are provided as a Source Data file.
