# Supplementary material for: A pulsatile release platform based on photo-induced imine-crosslinking hydrogel promotes scarless wound healing
Source: Nat Commun. 2021 Mar 15;12:1670. doi: 10.1038/s41467-021-21964-0 (PMC7960722; doi:10.1038/s41467-021-21964-0)
Supplement: Supplementary file 2 — Reporting Summary [file 41467_2021_21964_MOESM2_ESM.pdf]

nature research

Corresponding author(s): *Double-blind peer review submissions: write DBPR and your manuscript number here instead of author names.*

Last updated by author(s): *YYYY-MM-DD*

Reporting Summary

Nature Research wishes to improve the reproducibility of the work that we publish. This form provides structure for consistency and transparency in reporting. For further information on Nature Research policies, see our Editorial Policies and the Editorial Policy Checklist.

Statistics

For all statistical analyses, confirm that the following items are present in the figure legend, table legend, main text, or Methods section.

n/a	Confirmed	
☐	☒	The exact sample size (n) for each experimental group/condition, given as a discrete number and unit of measurement
☐	☒	A statement on whether measurements were taken from distinct samples or whether the same sample was measured repeatedly
☐	☒	The statistical test(s) used AND whether they are one- or two-sided<br>*Only common tests should be described solely by name; describe more complex techniques in the Methods section.*
☐	☒	A description of all covariates tested
☐	☒	A description of any assumptions or corrections, such as tests of normality and adjustment for multiple comparisons
☐	☒	A full description of the statistical parameters including central tendency (e.g. means) or other basic estimates (e.g. regression coefficient) AND variation (e.g. standard deviation) or associated estimates of uncertainty (e.g. confidence intervals)
☐	☒	For null hypothesis testing, the test statistic (e.g. F, t, r) with confidence intervals, effect sizes, degrees of freedom and P value noted<br>*Give P values as exact values whenever suitable.*
☒	☐	For Bayesian analysis, information on the choice of priors and Markov chain Monte Carlo settings
☒	☐	For hierarchical and complex designs, identification of the appropriate level for tests and full reporting of outcomes
☒	☐	Estimates of effect sizes (e.g. Cohen's d, Pearson's r), indicating how they were calculated

Our web collection on statistics for biologists contains articles on many of the points above.

Software and code

Policy information about availability of computer code

Data collection	TopSpin for 1HNMR; Gen5 for microplate reader; RheoWin for rheometer; Gotech materials testing software for mechanical tests; Living Image® software for in vivo imaging system; Ti2 Control Ver1.0.2 for microscope; SmartSEM for Field-emission Scanning Electron Microscope
Data analysis	Excel2010; originlab2016

For manuscripts utilizing custom algorithms or software that are central to the research but not yet described in published literature, software must be made available to editors and reviewers. We strongly encourage code deposition in a community repository (e.g. GitHub). See the Nature Research guidelines for submitting code & software for further information.

Data

Policy information about availability of data

All manuscripts must include a data availability statement. This statement should provide the following information, where applicable:

- Accession codes, unique identifiers, or web links for publicly available datasets
- A list of figures that have associated raw data
- A description of any restrictions on data availability

For this study, we will make our data available to the scientific community, which will avoid unintentional duplication of research. All the research data will be shared openly and in a timely manner in accordance with the most recent NIH guidelines (http://grants.nih.gov/grants/policy/data_sharing/).

Field-specific reporting

Please select the one below that is the best fit for your research. If you are not sure, read the appropriate sections before making your selection.

[x] Life sciences [ ] Behavioural & social sciences [ ] Ecological, evolutionary & environmental sciences

For a reference copy of the document with all sections, see nature.com/documents/nr-reporting-summary-flat.pdf

Life sciences study design

All studies must disclose on these points even when the disclosure is negative.

Sample size	Sample size was determined based on mean and standard deviation data from our preliminary tests.
Data exclusions	No data was excluded from the analyses.
Replication	All attempts at replication were successful. FESEM, phase contrast imaging, epifluorescence imaging and immunological staining experiments have been replicated at least three times independently to ensure reproducibility of results.
Randomization	All experimental units were randomly allocated across the treatment groups.
Blinding	The investigators were blinded to all group allocation during data collection and/or analysis.

Reporting for specific materials, systems and methods

We require information from authors about some types of materials, experimental systems and methods used in many studies. Here, indicate whether each material, system or method listed is relevant to your study. If you are not sure if a list item applies to your research, read the appropriate section before selecting a response.

Materials & experimental systems

n/a	Involved in the study	
[ ]	[x]	Antibodies
[ ]	[x]	Eukaryotic cell lines
[x]	[ ]	Palaeontology and archaeology
[ ]	[x]	Animals and other organisms
[x]	[ ]	Human research participants
[x]	[ ]	Clinical data
[x]	[ ]	Dual use research of concern

Methods

n/a	Involved in the study	
[x]	[ ]	ChIP-seq
[x]	[ ]	Flow cytometry
[x]	[ ]	MRI-based neuroimaging

Antibodies

Antibodies used	Anti-phospho-TGFβR1(P-S165) was from Aviva Systems Biology (OAAI00768, polyclonal, 1:200 dilution). Anti-phospho-SMAD2 (S465, S467) and anti-phospho-SMAD3 (S423, S425) were from Invitrogen (44-244G and 44-246G, polyclonal, 1:100 dilution for both). Anti-alpha-smooth muscle actin was from DAKO (M0851, clone 1A4, 1:100 dilution). Anti-F4/80 was from AbD Serotec (MCA497GA, clone Cl:A3-1, 1:200 dilution). Anti-CD4+ was from Affymetric eBioscience (14-9766-82, clone 4SM95, 1:600 dilution).
Validation	anti-phopho-TGFβR1 (Aviva Sym Bio OAAI00768) has been validated against human, rat and mouse, with applications for WB, ELISA, IP, IHC. Anti-phospho-SMAD2 (Invitrogen 44-244G) has been validated against human and mouse, with applications for ChIP, ICC, IF, IHC, WB, IHC. Anti-phospho-SMAD3 (Invitrogen 44-246G) has been validated against human and mouse, with applications for ICC, IF, WB, IHC. Anti-alpha-smooth muscle actin (DAKO, M0851) has been validated against human, with application for IHC. Anti-F4/80 (AbD Serotec, MCA497GA) has been validated against mouse, with applications for Flow, IHC, ELISA, WB, IF. Anti-CD4 (Affymetric eBioscience, 14-9766-82) has been validated against mouse, with applications for IHC, WB, ICC, IF, Flow.

Eukaryotic cell lines

Policy information about cell lines

Cell line source(s)	Mouse primary keratinocytes were isolated from newborn mouse skin. Caco-2 and Hela cells were purchased from ATCC.
Authentication	Caco-2 and Hela cell lines were obtained directly from ATCC. Keratinocytes are primary cell culture from mouse newborn skin.
Mycoplasma contamination	All the cells were tested and not contaminated with mycoplasma.

Commonly misidentified lines (See ICLAC register)	No commonly misidentified cell line was used in this study.

Animals and other organisms

Policy information about studies involving animals; ARRIVE guidelines recommended for reporting animal research

Laboratory animals	CD1 male mice aged 6-8 weeks; nude mice aged 6-8 weeks, adult New Zealand White female rabbits; male Yorkshire porcine (25-30kg). All mice used in this study were bred and maintained at the ARC (animal resource center) of the University of Chicago in accordance with institutional guidelines. All the mice were housed under pathogen-free conditions in the ARC (Animal Resources Center) at the University of Chicago under a 12 hour light-dark cycle. Housing facility maintains a temperature at 70-73 degrees (average 72) and humidity at 40-50% (average 44%)
Wild animals	The study did not involve wild animals.
Field-collected samples	The study did not involve samples collected from field.
Ethics oversight	All the experimental procedures on live animals (mouse, rabbit, and pig) were carried out in line with the Institutional Animal Care and Use Committee (IACUC) approved protocols of the Animal Care Center at the University of Chicago and Changhai Hospital, Shanghai, China.

Note that full information on the approval of the study protocol must also be provided in the manuscript.
